# Recent advances in the management of malignant pheochromocytoma and paraganglioma: focus on tyrosine kinase and hypoxia-inducible factor inhibitors

**DOI:** 10.12688/f1000research.13995.1

**Published:** 2018-07-30

**Authors:** Rodrigo Toledo, Camilo Jimenez

**Affiliations:** 1 Gastrointestinal and Endocrine Tumours Group, Vall d’Hebron Institute of Oncology, Barcelona, Spain; 2 Department of Endocrine Neoplasia and Hormonal Disorders, The University of Texas MD Anderson Cancer Center, Houston, Texas, USA

**Keywords:** Metastatic Pheochromocytoma, Metastatic paraganglioma, tyrosine kinase inhibitors, HIF inhibitors

## Abstract

Inactivating mutations of the succinate dehydrogenase subunit B ( *SDHB*) gene and the subsequent stabilization and activation of the hypoxia-inducible factor 2-alpha (HIF2α) unit are recognized hallmarks associated with the development of metastatic pheochromocytomas and paragangliomas (MPPG). Despite this discovery, the development of systemic therapies for patients with MPPG has been very slow. The rarity of the disease, the lack of preclinical animal models, and the impracticable development of large clinical trials has hindered the therapeutic progress for MPPG. Chemotherapy and low-specific activity ^131^meta-iodo-benzyl-guanidine (MIBG) (manufactured by simple isotope exchange methodology) led to positive clinical responses in about a third of patients. Molecular targeted therapies were introduced into oncological clinical practice at the beginning of the 21st century. These therapies have been demonstrated to be effective for patients with cancers that previously exhibited limited responses to systemic chemotherapy, such as kidney and thyroid carcinomas and pancreatic neuroendocrine tumors. The pathogenesis of MPPG overlaps in some way with the pathogenesis of kidney, medullary thyroid, and pancreatic neuroendocrine carcinomas, providing scientific support to explore molecular targeted therapies such as tyrosine kinase and HIF inhibitors.

## Introduction

Pheochromocytomas and paragangliomas (PPG) are rare neuroendocrine tumors originating in the paraganglia. Pheochromocytomas originate in the adrenal medulla, and paragangliomas originate in the extra-adrenal paraganglia. Most of these tumors secrete excessive amounts of catecholamines that predispose patients to cardiovascular and gastrointestinal morbidity ^1, 2^. About 15% to 20% of these tumors are metastatic, leading to a decreased overall survival ^3^. There are no histological, genetic, or molecular markers that could distinguish between benign and malignant disease and subsequently the diagnosis of malignancy relies exclusively on the presence of metastases ^4^; unfortunately, by then, the disease is usually advanced ^5^. In fact, only 50% to 60% of patients with metastatic pheochromocytomas and paragangliomas (MPPG) are still alive 5 years after the discovery of metastases ^6^. Metastases usually involve the lymph nodes (80%), the skeletal tissue (71%), the liver (50%), and the lungs (50%) ^7^. Whereas some patients succumb to the metastatic tumor burden, others may die because of complications derived from the excessive secretion of catecholamines ^8^. Currently, there are no systemic therapies approved by the European Medicines Agency or the US Food and Drug Administration (FDA) for patients with MPPG. Treatment options are limited to chemotherapy and low-specific activity ^131^meta-iodo-benzyl-guanidine (MIBG) and usually fail to produce a prolonged remission ^9, 10^. Furthermore, toxicity associated with chemotherapy and radiopharmaceutical agents cannot be underestimated ^11^. This scenario illustrates that the identification of clinically effective medications to treat ****MPPG is perhaps the most important unmet clinical need.

## Molecular pathogenesis of MPPG

The molecular pathogenesis of a substantial number of MPPG was elucidated in the early 2000s, when germline mutations of the succinate dehydrogenase subunit B ( *SDHB*) gene were identified ^12, 13^. Since then, the link between *SDHB* loss and increased risk for MPPG has been validated by several independent studies. It has been determined that about 30% to 40% of patients with MPPG carry a germline mutation of the *SDHB* gene ^14, 15^. These mutations prevent the oxidative catabolism of succinate to fumarate and electron transportation through the internal mitochondrial membrane. Consequently, accumulation of succinate acts as an oncometabolite, leading to stabilization and activation of hypoxia-inducible factors (HIFs), mainly the HIF2-alpha (HIF2α) unit ^16, 17^. Increased expression of HIF2α-targeted genes such as the vascular endothelial growth factors (VEGFs) and the platelet-derived growth factor beta (PDGF-β) and their receptors is observed in *SDHB* MPPG as well as many apparently sporadic tumors ^18^. In addition, genes involved in glucose metabolism, such as the hexokinase 2 and lactate dehydrogenase genes, are also upregulated ^18^. Activation of all of these genes leads to abnormally increased angiogenesis and cell growth, decreased apoptosis, and increased glucose uptake ^19^.

## Challenges in the discovery of new medications to treat MPPG

Although the genetic causes of many MPPG (mainly *SDHB* mutations) and the molecular events leading to the metastatic transformation of chromaffin cells (stabilization and activation of HIF2α, DNA hypermethylation) ^20^ were determined several years ago, the development of therapeutics against MPPG has been very slow for three main reasons: (a) difficulty of patient enrollment in large clinical trials, (b) lack of preclinical animal models, and (c) lack of efficient, targeted drugs. Given the rarity of MPPG (estimated incidence is less than one per million people per year), it is almost impossible to have multiple clinical trials testing a variety of drugs or drug combinations concomitantly. Several knockout mouse models for *SDHB* and other pheochromocytoma- and paraganglioma-related genes leading to activation of HIF2α (that is, von Hippel-Lindau and the mitochondrial enzymatic complex II subunit D genes) have not been demonstrated to mimic the human phenotype ^21, 22^. The lack of a reliable preclinical animal model is a major drawback that has impaired the screening of available drugs and drug combinations. Subsequently, the design of effective clinical trials relies mainly on clinical observations and increases the risk of wasting time and effort on trials that yield little or no benefit for patients with MPPG ^23, 24^. Moreover, the lack of animal models makes it very difficult to identify mechanisms of resistance that would enable the design of trials that combine therapies that could concomitantly or sequentially tackle escape pathways, prolonging clinical benefits. Therefore, clinical scientists and pharmaceutical research have prioritized their efforts on the few most promising medications to ensure sufficient patient enrollment. These difficulties have resulted in very slow progress and circumscribed therapeutic improvements.

## Tyrosine kinase inhibitors under evaluation in clinical trials

**Figure 1.**
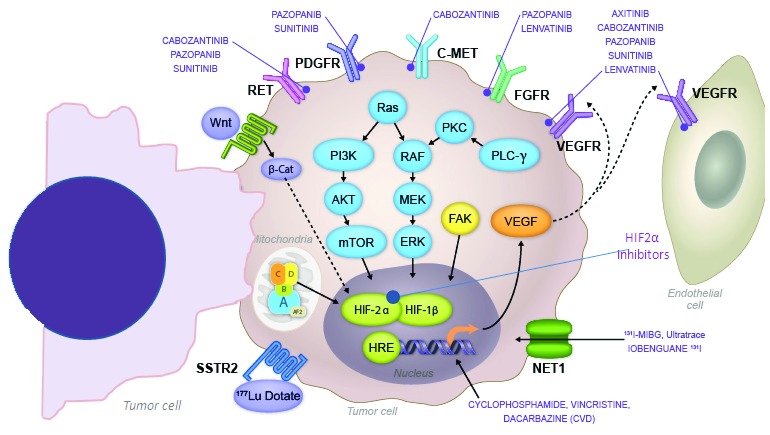
Pharmacodynamics of tyrosine kinase and hypoxia-inducible factor 2α inhibitors under evaluation in clinical trials for patients with metastatic pheochromocytomas and paragangliomas. This figure includes information on the mechanism of action of systemic chemotherapy and radiopharmaceutical agents: ^131^meta-iodo-benzyl-guanidine (MIBG) and ^177^Lu-DOTATATE.

Several tyrosine kinase inhibitors (TKIs), including axitinib, cabozantinib, lenvatinib, pazopanib, and sunitinib, are currently under evaluation in phase II clinical trials ( www.ClinicalTrials.gov). These agents have in common their capacity to block the activation of the VEGF receptors (VEGFRs), preventing angiogenesis and cell growth ^25^ ( Figure 1). In addition, TKIs can inhibit other tyrosine kinase receptors that are universally involved in processes such as cancer cell growth, tumor spread, and development of resistance ^26, 27^ ( Figure 1). Compelling positive results derived from phase III clinical trials have led to their approval by regulatory agencies for the treatment of malignancies such as kidney, thyroid, and pancreatic neuroendocrine carcinomas. Of interest, the pathogenesis of these tumors frequently overlaps with the pathogenesis of MPPG, supporting the development of clinical trials for MPPG. A historical description of preliminary clinical findings in MPPG treated with these medications follows.

### 1. Sunitinib

Sunitinib was the first TKI described as a possibly effective treatment for patients with MPPG. Sunitinib was approved for the treatment of advanced kidney cancer on the basis of the impressive results derived from a phase III clinical trial ^28^. In 2008, two simultaneous case reports described potential benefits derived from sunitinib. In a patient with MPPG in the context of von Hippel-Lindau disease, sunitinib was associated with tumor size reduction and blood pressure and pain control. The decision to study sunitinib in this patient was supported by the demonstration of a very high expression of VEGF and PDGFRB-1 in the removed primary tumor and the simultaneous presentation of progressive multifocal kidney cancer for which sunitinib was indicated ^29^. In another case report, the discovery of benefits derived from sunitinib was accidental. The patient presented with a large unresectable mass suspicious of kidney cancer; the patient received sunitinib and the tumor became resectable. Surprisingly, histological evaluation confirmed a paraganglioma ^30^. Over time, several MPPG patients who were not candidates or responsive to chemotherapy/MIBG received sunitinib. In a retrospective intention-to-treat analysis of 17 patients who received sunitinib, 47% exhibited partial responses and disease stabilization with blood pressure control despite catecholamine excess. Positive responses were noticed in carriers of *SDHB* mutations as well as patients with apparently sporadic tumors. Progression-free survival was only 4.1 months; 23.5% of patients discontinued therapy because of adverse events such as overwhelming fatigue, pain exacerbation, hand and foot syndrome, and cardiovascular disease (severe hypertension and syncope) ^31^. Two phase II clinical trials with sunitinib are ongoing ( www.ClinicalTrials.gov).

### 2. Pazopanib

The mechanism of action of pazopanib overlaps with that of sunitinib; like sunitinib, pazopanib is approved for the treatment of patients with kidney cancer. Some studies in kidney cancer previously suggested that pazopanib could be an easier medication to tolerate than sunitinib ^32, 33^. A phase II clinical trial for MPPG with pazopanib was subsequently developed. The primary endpoint of the trial was best objective response rate. Intervention was pazopanib 400 mg daily for 2 weeks followed by dose titration to 800 mg daily. The trial recruited seven patients. Only one patient exhibited a confirmed partial response that lasted for about 2 years. Several patients had overwhelming side effects: 17% had grade 3–4 diarrhea, hematuria, headaches, and fatigue, and 50% had severe hypertension ^34^. Toxicity was more obvious after doubling the dose of pazopanib. The trial was terminated because of lack of recruitment.

### 3. Axitinib

Axitinib is a pure anti-angiogenic medication. As it only blocks the VEGFR, it may be associated with fewer adverse events when compared with sunitinib and pazopanib ^35^. Axitinib is also approved for the treatment of kidney cancer. However, experience with axitinib in patients with kidney cancer has demonstrated that hypertension might be a difficult problem to face during treatment ^36^. A phase II clinical trial for MPPG was developed. The trial recruited 11 patients. The primary endpoint was objective response rate. Intervention was axitinib 5 mg twice dose with dose titration to 7.5–10 mg twice a day; 36% of patients achieved a partial response. Severe hypertension was noticed in 82% of patients and dose could not be titrated up. Conversely, all patients required dose reduction ^37^. The trial is currently closed for recruitment.

### 4. Cabozantinib

Cabozantinib is perhaps the most potent anti-angiogenic medication currently approved for the treatment of cancer ^38^. In addition to inhibiting the VEGFRs, cabozantinib inhibits the c-met receptor pathways which are involved in tumor growth and spread and the development of resistance to anti-angiogenesis ^38^. Cabozantinib is approved for the treatment of medullary thyroid and kidney cancers ^39, 40^ and seems to be more effective than sunitinib in advanced kidney cancer ^41^. A phase II clinical trial with cabozantinib is ongoing. The trial is expected to recruit 22 patients. The primary endpoint is objective response rate. Intervention is cabozantinib 60 mg daily. Unlike the previously described clinical trials, the trial with cabozantinib allows dose titration down to 40 and 20 mg, as clinical observations in patients with other cancers have demonstrated positive responses despite the dose reduction. Preliminary results derived from 14 patients who received cabozantinib reveal that 93% of them have exhibited partial responses or disease stabilization with some degree of regression ^42^. To date, the clinical trial has not reported grade 4 or 5 adverse events. Two patients presented with grade 3 adverse events; these events were an asymptomatic elevation of pancreatic enzymes and a rectal fistula that was corrected with surgery. Hypertension, mainly grade 1, has been noticed in 40% of patients ^42^. The trial includes an exploratory branch for patients with predominant bone metastases. The trial is actively recruiting patients.

### 5. Lenvatinib

Lenvatinib is another potent anti-angiogenic medication. It also inhibits the fibroblast growth factor receptor pathway. Lenvatinib is approved for the treatment of advanced thyroid cancer of follicular origin ^43^ and has demonstrated clinical effectiveness in medullary thyroid cancer ^44^. Lenvatinib in combination with everolimus is also approved for the treatment of kidney cancer ^45^. Isolated clinical experience suggested that this medication may have a positive impact on MPPG. A phase II clinical trial with lenvatinib is looking at objective response rate as the primary endpoint. This trial recently initiated recruitment ( www.ClinicalTrials.gov).

## Tyrosine kinase inhibitors and cardiovascular events in patients with MPPG

Preliminary results of several clinical trials show that several TKIs with mainly anti-angiogenic activity may cause anti-tumor effects in a substantial number of patients with MPPG ^31, 34^. However, effectiveness has been undermined by cardiovascular events ^34^. It is important to recognize that MPPG are indeed more difficult to treat than other cancers given the endocrine nature of the disease. Most patients with MPPG are found with a large tumor burden ^3^. Furthermore, the majority of these tumors secrete excessive amounts of catecholamines, mainly noradrenaline. Therefore, patients with MPPG have an elevated risk for severe cardiovascular disease upon exposure to systemic therapies such as TKIs. Hypertension and cardiovascular disease may be caused by a combination of medication, direct cardiovascular toxicity (that is, inhibition of nitric oxide synthesis) ^46^, or the rapid and massive release of catecholamines once the tumor destruction starts. Guidelines on how to treat MPPG are not existent. Nevertheless, treatment with alpha- and beta-blockers should be offered to all patients with catecholamine-secreting PPG ^1, 47^. Alpha-blockers should be started first and titrated as soon as possible in order to achieve orthostatism and allow the initiation of the beta-blockers to protect the heart ^1^. Alpha- and beta-blockers could be titrated to very high doses (that is, doxazosin 16 mg daily; propranolol 640 mg daily) ^48, 49^; nevertheless, concerns related to high-dose toxicity and lack of effectiveness have been raised. Therefore, patients frequently need the addition of other anti-hypertensive drugs such as calcium channel blockers, angiotensin-converting enzyme inhibitors, angiotensin receptor blockers, labetalol, hydralazine, or catecholamine synthesis inhibitors (metyrosine) or a combination of these ^50^. In fact, studies have shown that patients with MPPG may need an average of four to six anti-hypertensive drugs in order to normalize their blood pressure. The clinical trials have shown that the doses of different TKIs must be carefully chosen. As suggested by the preliminary results of the cabozantinib phase II study, it is always better to choose a starting dose that is clearly associated with clinical benefits and that allows dose titration down, preserving effectiveness. This approach may allow one to later titrate the dose up once an anti-tumor effect is achieved, as catecholamine secretion may have already decreased by then. In the meantime, anti-hypertensive doses should be adjusted accordingly. Acute complications are not uncommon, and treating physicians and principal investigators must be familiar with the treatment of hypertensive crisis with medications such as nitroprusside, nicardipine, and esmolol. It is also important to remember that patients with MPPG may have easier-to-control blood pressure after primary tumor resection (if possible) because of a decreased catecholamine surge ^51^. Surgery of the primary tumor and solitary metastasis should be considered before any systemic therapy is instituted.

## Tyrosine kinase inhibitors and other drug-related adverse events

Patients who receive TKIs frequently complain of constitutional symptoms, hand and foot syndrome, pain exacerbation, and gastrointestinal irritability among other symptoms. Treating physicians must be familiar with these potential side effects and beforehand should establish therapeutic interventions that could prevent exacerbation of these symptoms to guarantee a successful treatment. These interventions may include—but are not limited to—the use of moisturizer lotions to prevent dryness, avoidance of overuse of hands and feet, and adjustment of analgesic doses before initiation of therapy to prevent pain exacerbation in patients with bone metastases. Constipation is an uncommon but sometimes difficult complication in patients with MPPG ^2^. TKIs frequently cause diarrhea. Although this adverse event could benefit patients with constipation, our clinical experience indicates that it is better to treat the constipation with other interventions before a molecular targeted therapy is started. Symptoms such as nausea, vomiting, and abdominal pain could be severe in constipated patients who receive targeted therapies ^52^; these symptoms may prevent patients from taking systemic therapy.

## Anti-angiogenic TKIs inhibit part of the HIF2α pathway

As described above, treatment of MPPG with anti-angiogenic TKIs can lead to partial responses and stable disease; however, such treatments may have limited long-term benefits, since patients developed subsequent resistance and progression ^31^. One plausible explanation for this is that while these drugs inhibit mainly the angiogenic pathway through VEGFRs, the observed progression and resistance may be related to the compensatory activation of HIF2α molecular pathways caused by hypoxia induced by blood vessel regression ^53^.

Robust molecular studies have identified HIF2α as one of the main oncogenic drivers of paraganglioma/pheochromocytoma (PPGL) ^54, 55^. In addition to *SDHB* mutations leading to HIF2α activation, mutations in the HIF2α-encoding gene ( *EPAS1*) have been identified and functionally characterized in PPGL ^56^. These validated findings, implicating the disruption of genes involved in the response to hypoxia, have spearheaded the initiation of therapeutic strategies to directly tackle HIF2α ( Figure 1).

## Development of first-in-class HIF2α inhibitors

For many years, transcription factors, including HIF2α, were considered undruggable, and pharmaceutical research focused mainly on the HIF pathway downstream (that is, VEGFR2). In 2009, the structure of a heterodimer between the HIF2α PAS (Per-ARNT-Sim) domains—which sensor oxygen and REDOX potential—and ARNT was solved by crystallography, enabling the identification of a large protein cavity located within the HIF2α PAS-B domain. Such a bulky cavity, which is extremely rare in proteins, was expected to accommodate small molecules and therefore to be successfully targeted. Based on the structure of the HIF2α:ARNT protein dimer, an extensive screening of small-molecule libraries has been performed and identified specific HIF2α inhibitors with potential for clinical development.

## HIF2α inhibitors show low toxicity and high clinical efficiency in human hypoxic tumors

HIF2α inhibitors have shown tumor inhibition in both *in vitro* and *in vivo* models of clear cell renal cell carcinomas (RCCs) associated with pseudo-hypoxia ^57^. The results of a clinical trial with a HIF2α inhibitor in patients with locally advanced or metastatic RCC that progressed with at least one prior systemic therapy revealed complete responses, partial responses, and stable disease in 2%, 12%, and 52% of heavily pretreated patients, respectively. Of interest, no patients discontinued treatment because of adverse events ^58^. Further studies are required to determine whether such promising HIF2α antagonists will be effective for the treatment of MPPG.

## Other molecular targeted therapies

In addition to the tyrosine kinase and HIF2α inhibitors, there are molecular targeted therapies that have or may have a positive impact in MPPG. These therapies include radiopharmaceutical medications such as high-specific activity MIBG and peptide receptor radionuclide therapy (PRRT). The final results of the phase II clinical trial with high-specific activity MIBG (Azedra ^®^) for MPPG that express the cell membrane catecholamine-uptake transporter are impressive; more than 90% of patients exhibit clinical benefits ^59^. This medication has received breakthrough therapy designation by the FDA, and the clinical results are currently under evaluation by this regulatory agency. PRRT was recently approved for the treatment of gastroenteropancreatic neuroendocrine tumors that express cell membrane somatostatin receptors ^60^. MPPG frequently express somatostatin receptors in the cell membrane; subsequently, these patients may benefit from PRRT ^61^. A phase II clinical trial with ^177^Lu-DOTATATE was recently activated ( www.ClinicalTrials.gov). As the focus of this article is on tyrosine kinase and HIF2α inhibitors, we will not discuss these therapies further.

## Conclusions

Observations derived from phase II clinical trials with tyrosine kinase and HIF inhibitors have revealed anti-neoplastic effects. However, it is important to recognize that MPPG are more challenging to treat when compared with other oncological conditions that are also treated with these medications. Their large tumor burden and their frequently overwhelming hormonal manifestations may lead to a high rate of adverse events. Clinical trials must be carefully designed and should actively involve clinicians familiar with the endocrine manifestations of MPPG. Results of several clinical trials with TKIs are still preliminary, and we cannot yet define the therapeutic role that these medications might have in MPPG. HIF2α inhibitors may target the core of the MPPG pathogenesis and, together with cabozantinib, are perhaps some of the most exciting medications to explore in MPPG. As expected, tyrosine kinase and HIF inhibitors could control but alone cannot cure advanced MPPG. Therefore, the research effort to understand the pathogenesis of MPPG must continue, as it is clearly helping to identify potential therapies. This effort will help us to recognize medications with unique and fundamental mechanisms of action that, when used alone or especially in combination, may finally help to conquer this orphan and devastating disease.
